# Concurrent Papillary Thyroid Carcinoma and Synovial Carcinoma of the Neck in an Adult Male

**Published:** 2019-01

**Authors:** Haissan Iftikhar, Shabbir Akhtar, Nasir Uddin

**Affiliations:** 1 *Department of Otolaryngology, Aga Khan University hospital, Karachi, Pakistan. *; 2 *Department of Pathology, Aga Khan University hospital, Karachi, Pakistan.*

**Keywords:** Head and Neck, Sarcoma neck, Thyroid cancer

## Abstract

**Introduction:**

Synovial sarcoma makes up 8–10% of all soft tissue sarcomas, and constitutes 3–10% of all sarcomas occurring in the head and neck region. It shows male predominance (3:2), and the mean age of presentation is 30 years.

**Case Report:**

A 51-year-old gentleman presented with right-sided neck swelling which had been progressively increasing in size for the past 2 years. A computed tomography (CT) scan revealed a large heterogeneously enhancing mass on the right side of the neck measuring 7.5 × 6.2 cm. Biopsy of an enlarged node revealed papillary thyroid carcinoma. The patient subsequently underwent total thyroidectomy with right neck dissection. Final histopathology revealed a papillary carcinoma of the thyroid, and the right-sided mass was shown to be monophasic synovial sarcoma.

**Conclusion:**

We present a case of a concurrent pathology of neck papillary thyroid carcinoma with monophasic synovial sarcoma. We experienced difficulty in diagnosis and misdirection due to raised C-reactive protein (CRP) levels, until final histopathology of the neck mass.

## Introduction

Synovial sarcoma makes up 8–10% of all soft tissue sarcomas, and constitutes 3–10% of all sarcomas occurring in the head and neck region (1). The usual site of a tumor in the head and neck region is the prevertebral space and the base of the skull; however, it can occur anywhere from the skull base to the retropharyngeal and parapharyngeal space to the anterior neck (1). It shows male predominance, and the mean age of presentation is 30 years (2-4). Our case is made special by the concurrent presence of two primary carcinomas of the neck, and the failure to detect synovial sarcoma on the initial biopsy of the lesion which was also masked by raised C-reactive protein (CRP). To the best of our knowledge, this case is the first reported case of papillary thyroid carcinoma and synovial sarcoma of the neck being diagnosed simultaneously.

## Case Report

**Fig 1:**
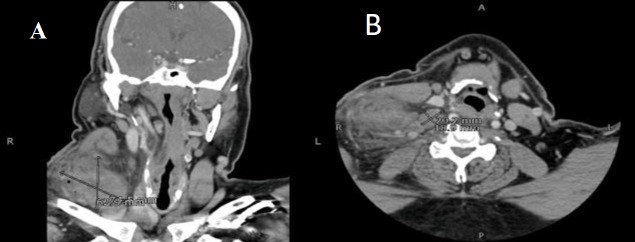
A: CT Scan (Coronal cut) large 7.5 x 6.2 cm mass in the right cervical region Fig-1B: CT Scan (Axial Cut) showing a 2 x 1.9 cm necrotic lymph node along with a mass laterally

A 51-year-old gentleman with a known history of hypertension presented with right-sided neck swelling, which had been progressively increasing in size for the past 2 years, along with dysphagia and odynophagia. The swelling was on the right side of the neck and was firm, non-mobile, and slightly tender to touch. It measured around 5 × 4 cm and was not adherent to the skin. Examination of the ear, nose, throat, and oral cavity was unremarkable, and flexible laryngoscopy revealed right vocal cord paresis. The patient had no complaint of voice change.A computed tomography (CT) scan of the head and neck with contrast was advised, and revealed a large heterogeneously enhancing solid lesion posterior to the right sternocleidomastoid muscle displacing the carotid space and medially extending inferiorly to the supraclavicular fossa. The mass measured 7.5×6.2 cm in size along its greatest dimensions (Fig1A,B). 

The patient underwent open biopsy with panendoscopy. The histopathology was inconclusive for malignancy; however, culture revealed S. aureus and Pseudomonas, with a raised CRP of 16.1 mg/dL. The patient was treated on the basis of an infectious etiology. The patient re-presented to us with complaints of rapidly increasing right-sided neck swelling for the past 3 months. A biopsy of an enlarged lymph node revealed papillary thyroid carcinoma.

The patient was electively taken for surgery and underwent total thyroidectomy with right-sided neck dissection. Intra-operatively, the right-sided lesion was extending from just above the clavicle to the base of the skull. It was adherent to the internal carotid artery and was involving the vagus and the accessory nerve, both of which had to be scarified during the excision of the lesion. The mass was also seen to be infiltrating the anterior-lying sternocleidomastoid and adherent to the internal jugular vein. In all, a radical neck dissection was carried out on the right side.

**Fig 2:**
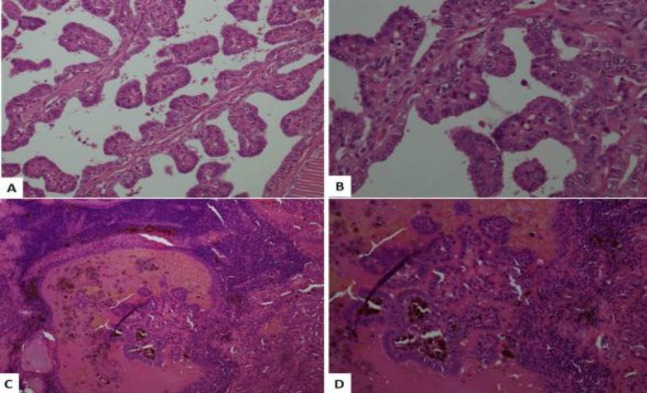
A,B: Low and high power examination reveal a papillary tumour with fibrovascular cores. The lining cells have overlapping nuclei with grooves and pseudo inclusions. C,D). Lymph node replaced by metastatic papillary thyroid carcinoma (H&E. low and high power magnification)

Histological examination of the thyroid gland showed multifocal papillary thyroid carcinoma involving both lobes with extrathyroidal spread. The tumor was composed of papillary structures with fibrovascular cores. The lining cells exhibited enlarged nuclei with focal grooves, pseudo-inclusions and overlapping (Fig.2 A,B). The tumor involved 8/13 neck lymph nodes (Fig.2 C,D), and was staged as pT3, N1. Histological examination of the separately sent 6×5×3.5 cm nodule showed a circumscribed cellular spindle cell neoplasm arranged in fascicles. 

**Fig 3:**
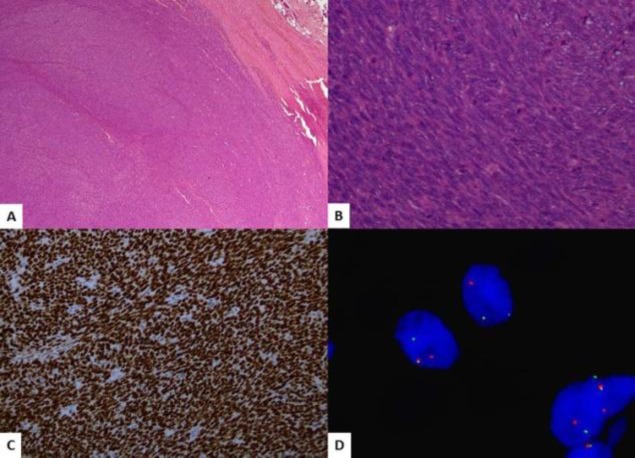
A, B: Circumscribed cellular spindle cell tumour arranged in fascicles. The cells have hyperchromatic nuclei. C). Diffuse strong nuclear positivity of TLE1 in tumour cells. D). FISH reveal break-apart signals in tumour cells

The tumor cells had hyperchromatic nuclei and eosinophilic characteristics (Fig3. A,B). Up to 11 mitoses/10 high-power fields (HPFs) were seen. The tumor cells were positive for Cytokeratin (CK) AE1/AE3,epithelial membrane antigen (EMA), Bcl2, CD99 and transducer-like enhancer of split 1(TLE1) (Fig.3C). 

The tumor cells were negative for desmin, anti-smooth muscle actin (ASMA), and S100. The morphological and immunohistochemical features were consistent with synovial sarcoma. Translocation SS18 was detected by fluorescence in situ hybridization (Fig.3D) Final diagnosis was conventional papillary thyroid carcinoma and monophasic synovial sarcoma of the neck.The patient was discharged in the subsequent days after neck drain removal and once stable. On follow-up fiberoptic laryngoscopy, right-sided vocal cord paralysis was seen, which was expected. Radiation and chemotherapy followed by radio-iodine ablation was advised after the case was discussed in the multidisciplinary tumor board meeting. The patient was disease free 6 months’ post-surgery and had started undergoing chemotherapy from another institute.

## Discussion

Despite its name, synovial sarcoma rarely involves the synovial membrane, but rather originates from mesenchymal pluripotent stem cells (1,2). Synovial sarcomas are commonly found around the para-articular region of the large joints commonly in the lower extremities (1,3). Synovial sarcoma most commonly presents as a painless mass, but can also present with pain, bleeding, or compressive symptoms due to its enlarging size (1). Our patient presented with a painless right cervical swelling associated with mild dysphagia to solids. Histopathologically synovial sarcoma has two variants: monophasic and biphasic. The biphasic tumor has both epithelial and spindle cells, whereas the monophasic variant has just one component and poses a diagnostic challenge (5).

Wide excision with clear margins is the mainstay of treatment, but this is not always possible given the close vicinity of the vital structures (1). Radiotherapy is therefore advised, and it is seen that patients with post-operative radiotherapy have a better survival (5). Although the role of chemotherapy is not well established, chemotherapy was associated with improved survival in one retrospective analysis (6). As lymph node metastasis is not commonly seen with synovial sarcoma (1), routine clearance of the neck nodes is not suggested. Enlarged lymph nodes or suspicious nodes should be sent for histopathology. There is a high recurrence rate of 60–90% in the first 2 years after local excision (1). The overall survival in patients with synovial sarcoma is reserved (7). Prognostic factors include the age, size and depth of the tumor at the time of excision (1). In our case, the patient had many poor prognostic factors, including being over 50 years of age, involvement of surrounding structures, concomitant papillary thyroid carcinoma, mass extending posteriorly in the neck, and the large size of the tumor in itself.

Papillary thyroid cancer has been discussed in detail in the literature, and we therefore do not touch on that topic here. Synovial sarcoma has been previously reported in a patient with papillary thyroid carcinoma (8). She initially had fibrosarcoma in the right supraclavicular region and had received external beam radiotherapy also covering the right lobe of thyroid. Seventeen years after radiotherapy, the patient developed papillary thyroid carcinoma in the right lobe, and developed synovial sarcoma 2 years later (8).

To the best of our knowledge, our patient is the first reported case of a simultaneous diagnosis of papillary thyroid carcinoma and monophasic synovial sarcoma.

## Conclusion

Malignancy cannot be definitely ruled out even after a negative fine-needle aspiration cytology (FNAC) and biopsy. We present a case of a concurrent pathology of neck papillary thyroid carcinoma with monophasic synovial sarcoma where we had difficulty in diagnosis and misdirection until final histopathology of the neck mass, owing to the raised CRP. Dual malignancy may present with raised inflammatory markers and should be considered unless proven otherwise.
